# High Repetition Rate UV versus VIS Picosecond Laser Fabrication of 3D Microfluidic Channels Embedded in Photosensitive Glass

**DOI:** 10.3390/nano8080583

**Published:** 2018-07-31

**Authors:** Florin Jipa, Stefana Iosub, Bogdan Calin, Emanuel Axente, Felix Sima, Koji Sugioka

**Affiliations:** 1 Center for Advanced Laser Technologies (CETAL), National Institute for Laser, Plasma and Radiation Physics (INFLPR), 409 Atomistilor, Magurele RO-77125, Romania; florin.jipa@inflpr.ro (F.J.); stefana.iosub@inflpr.ro (S.I.); bogdan.calin@inflpr.ro (B.C.); emanuel.axente@inflpr.ro (E.A.); 2 RIKEN Center for Advanced Photonics, 2-1 Hirosawa, Wako, Saitama 351-0198, Japan

**Keywords:** picosecond laser processing, 3D microfluidic channels, photosensitive glass

## Abstract

Glass is an alternative solution to polymer for the fabrication of three-dimensional (3D) microfluidic biochips. Femtosecond (fs) lasers are nowadays the most promising tools for transparent glass processing. Specifically, the multiphoton process induced by fs pulses enables fabrication of embedded 3D channels with high precision. The subtractive fabrication process creating 3D hollow structures in glass, known as fs laser-assisted etching (FLAE), is based on selective removal of the laser-modified regions by successive chemical etching in diluted hydrofluoric acid solutions. In this work we demonstrate the possibility to generate embedded hollow channels in photosensitive Foturan glass volume by high repetition rate picosecond (ps) laser-assisted etching (PLAE). In particular, the influence of the critical irradiation doses and etching rates are discussed in comparison of two different wavelengths of ultraviolet (355 nm) and visible (532 nm) ranges. Fast and controlled fabrication of a basic structure composed of an embedded micro-channel connected with two open reservoirs, commonly used in the biochip design, are achieved inside glass. Distinct advantages such as good aspect-ratio, reduced processing time for large areas, and lower fabrication cost are evidenced.

## 1. Introduction

Microfluidic systems typically consisting of three-dimensionally (3D) embedded channels connected to open micro-reservoirs are useful tools for many biological and medical studies, since they are basic elements for biochips such as lab-on-a-chip devices and micro-total-analysis-systems that can perform reaction, detection, analysis, separation, and synthesis of biochemical materials with high-efficiency, high-speed, high-sensitivity, low reagent consumption, and low waste production [1,2]. The unique 3D geometries offer flexibilities and specific functionalities for fabrication of the biochips [3,4] or even organs-on-a-chip systems [5]. Such microfluidic devices for biomedical applications are generally fabricated based on PDMS with casting technologies [6,7]. However, although they exhibit indubitable advantages such as biocompatibility, good optical quality, and easy to use, some drawbacks including non-reusability, and adsorption of organic compounds, as well as the requirement of multiple stacking and sealing processes in the fabrication procedure push us to find an alternative [8]. During the last decade, femtosecond (fs) laser fabrication has proven to be a powerful tool for 3D internal modification of transparent glass materials and fabrication of embedded channels [9,10,11,12]. Thus, glass is a good alternative to PDMS for specific biological applications, which allow creating robust, easy to clean and reusable devices. Among many glasses, Foturan is one of the most suitable materials for fabrication of microfluidic systems. Foturan is a photosensitive glass with photoreactive properties due to the addition of a photoactive agent (photo-sensitizer) and metal ions (nucleation agent) in the glass matrix. The photoactive agent is cerium (<0.04 wt% Ce_2_O_3_), while the nucleation ion is silver (0.05–0.15 wt% Ag_2_O) [13]. Unlike other transparent glasses these agents allow this glass to be processed in a 3D manner by space selective control of the precipitation process [14]. Photoactivation takes place at wavelengths shorter than 350 nm, and then a successive annealing treatment induces silver clustering that converts a latent image of irradiated region into an observable one. During the thermal treatment, a metasilicate crystalline phase is grown around the formed silver clusters which, by an isotropic chemical etching, can be selectively removed to create 3D hollow micropatterns in the glass matrix [15]. Masuda et al. used a high-intensity fs laser emitting light of 150 fs pulse width at 775 nm wavelength, 1 kHz repetition rate and 0.4 μJ, to fabricate complex 3D microfluidic structures inside Foturan glass with a high spatial resolution [16]. In this case, the photo-reaction of near-infrared fs pulses with glass takes place by two-step excitation of electrons with three photon absorptions each, resulting in a six-photon process [17]. 

This technique enabling the fabrication of 3D microfluidic structures has been termed femtosecond laser-assisted etching (FLAE). FLAE of Foturan glass was then applied to fabricate specific biochips called nano-aquariums for monitoring of continuous motion of *Euglena gracilis* [18] and evaluation of gliding mechanism of *Phormidium* cyanobacteria [19]. Meanwhile, picosecond (ps) lasers, which are also categorized as ultrafast lasers, are becoming more common tools for practical use due to higher power and higher reliability as compared to fs lasers. At this time-scale, the deposition of laser energy is still typically faster than the electron-phonon coupling processes (which are material-dependent), enabling the minimization of heat-affected zone and, thus, high-quality micro- and nano-fabrication [20]. In addition, the high peak power (*P*_peak_ = *E*/*τ*, *E*—pulse energy, *τ*—pulse duration) can induce nonlinear absorption processes in materials which do not absorb the laser wavelength, thus allowing the processing not only of the surface, but also of the inside of transparent materials similarly to the fs lasers [9,10]. Therefore, the ps laser may be able to replace the fs lasers for 3D microfabrication of Foturan glass, which is beneficial in terms of the high throughput process.

Veiko et al. have demonstrated the possibility to modify Foturan glass by a ps Nd:YAG laser with a pulse width of 30 ps at 532 nm wavelength and a repetition rate of 10 Hz [21]. A mechanism based on two-photon absorption was proposed. Thus, microstructures attributed to the phase transition from amorphous to crystalline inside the Foturan glass-ceramic material were fabricated by means of local laser modification followed by subsequent thermal treatment. It has been further shown that using the same laser source it is possible to create 3D channels in bulk by hydrofluoric acid (HF) etching of crystallized regions developed by thermal treatment using a CO_2_ laser [22]. Even though the proof of concept was demonstrated, the potential of ps lasers for the structuring and fabrication of 3D embedded channels in Foturan glass is still not fully explored and many challenges remained unmet.

In this study we demonstrate the successful fabrication of 3D microfluidic structures in Foturan glass by high repetition rate ps laser-assisted etching (PLAE) using either the second or third harmonics (visible (VIS) 532 nm or ultraviolet (UV) 355 nm wavelengths) of a Nd:YVO_4_ laser. Critical irradiation doses and etching ratios were examined for both wavelengths to optimize the experimental conditions of PLAE. Controlled fabrication of the microfluidic structures consisting of an embedded channel connected with two open micro-reservoirs is achieved for both cases. Our study evidenced that transparent materials processing with high repetition rate ps laser pulses based on multi-photon absorption could be a viable alternative to classical fs micro-fabrication. More importantly, large-area 3D micro/nanofabrication with considerably reduced processing time and production costs will offer great advantages for manufacturing with high repetition rate ps laser pulses.

## 2. Materials and Methods

Foturan used in this study is a photo-structurable glass ceramic manufactured by Schott North America Inc., Corporate Office, Elmsford, NY, USA. It is a photo-sensitive alkali-aluminosilicate glass material consisting of SiO_2_ (75%–85%), Li_2_O (7%–11%), K_2_O and Al_2_O_3_ (3%–6%), Na_2_O (1%–2%), ZnO (0%–2%), Sb_2_O_3_ (0.2%–0.4%), Ag_2_O (0.05%–0.15%), and Ce_2_O_3_ (0.01%–0.04%). In our experiments, we cut Foturan glass wafers to 10 × 10 × 2 mm^3^ dimensions to create 3D microfluidic structures. Prior to use, the samples were successively cleaned in baths of acetone, alcohol, and deionized water.

**Figure 1:**
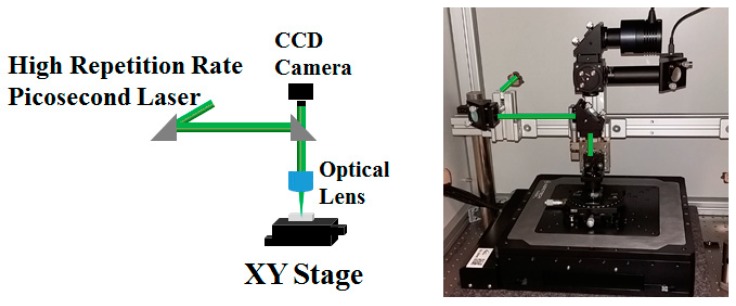
Scheme and photo of the workstation used for high repetition rate ps laser irradiation of Foturan glass.

The laser direct writing was conducted by a ps laser beam (Lumera, Coherent) delivering pulses of duration below 10 ps at 500 kHz repetition rate, coupled with a customized workstation (Figure 1). The second (532 nm—VIS) and the third (355 nm—UV) harmonics of a Nd:YVO_4_ laser were used in experiments with laser power ranging from tens of mW for critical doses up to 500 mW for material processing using VIS wavelength and from values below 1 mW for critical doses up to 10 mW for structuring in UV. For both wavelengths, the scanning speed was varied from 0.1 mm/s to 1 mm/s in order to determine the optimum value of interline spaces which is an important parameter for a line-by-line scanning process. The beam was focused using an aspheric lens of 15 mm focal length producing a circular focal spot of 4 μm in diameter. The entire writing process was monitored with a CCD camera, which was also used to control the focusing position on the sample surface and inside the volume. The samples were placed on an X-Y motorized translation stage (PlanarDL, Aerotech Inc., Pittsburgh, PA, USA) with computer-control which provided 200 mm travel range on each axis with ±500 nm step accuracy and ±100 nm precision. In addition, the stage has the ability to trigger the laser firing for precise synchronization, increasing thus the accuracy of the imprinted pattern.

The exposed samples were then annealed in a furnace (model MTF M1238-250 from Carbolite Gero Limited, Hope Valley, UK) controlled with the following program: heating with a slope of 5 °C/min up to 500 °C, then keeping the temperature constant for 1 h in order to grow Ag nanoparticles, increasing the temperature again with a slope of 3 °C/min to 605 °C, and keeping the temperature constant for another hour to obtain the crystalline phase of Li metasilicate. The process was followed by chemical etching in 8% HF solution under ultrasonic condition. During the etching, the crystalline phase grown around Ag nanoparticles was selectively removed. Profilometry analysis was carried out with a stylus profiler XP2 from Ambios Technology, 0.01 mm/s speed and 1 mm working distance. Optical interrogation of the samples was performed in transmission mode with a microscope, model DM4000 B Led from Leica Microsystems, Wetzlar, Germany. Scanning electron microscopy studies were employed with an FEI Co. microscope, model Inspect S, Hillsboro, OR, USA.

## 3. Results

### 3.1. Critical Dose Evaluation

In a first step, the experimental procedure was devoted to the evaluation of the critical dose *D*_c_ for each irradiation wavelength, in order to find optimum parameters for laser processing. By using a model proposed by Fuqua et al. [23] as the critical laser fluence *F*_c_, the critical irradiation dose can be expressed as:

(1) $$D_{c}=F_{c}^{m}\times N$$

*N* is the number of laser pulses necessary to induce photoreaction and Ag nucleation and *m* represents the number of photons in the multiphoton absorption process for electron generation. *F*_c_ is defined as the lowest fluence at which a sufficiently high density of nuclei (Ag nanoclusters) is able to form an interconnected network of the metasilicate crystalline phase in photo-structurable glass by the thermal treatment for selective removal. Here, the density of nuclei *ρ* generated at a fluence of *F* can be expressed by *ρ* = *K* × *F^m^* × *N* (*K* is a proportionality constant).

The determination of UV and VIS critical doses was performed by irradiating Foturan glass surface with different laser powers and exposure times. For fixed power values, several exposure times from 0.5 to 3 s were applied, corresponding to 2.5 × 10^5^ to 15 × 10^5^ pulses, resulting in the accumulation of different laser doses in distinct areas. The exposure reproducibility was demonstrated by generating a matrix of identical patterns for each area with a separation distance of 10 μm. This approach allowed easier optical evaluation of the samples and an improved accuracy in *D*_c_ evaluation.

**Figure 2:**
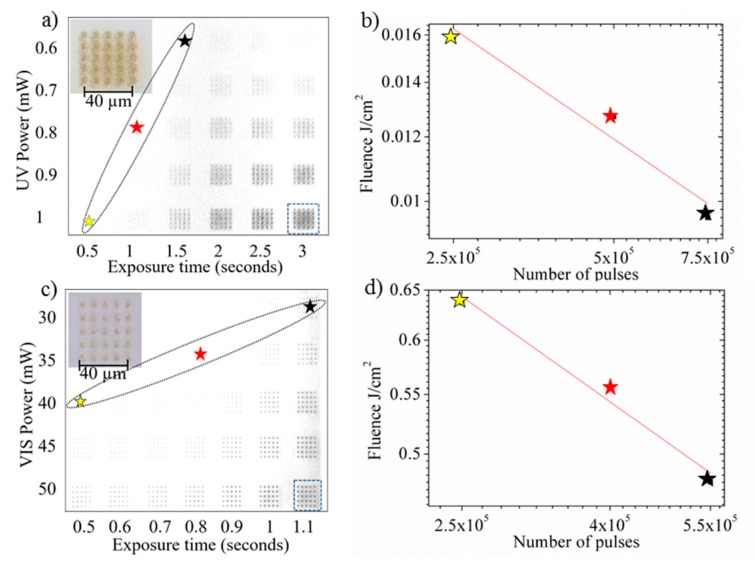
Identification of threshold irradiation parameters for ps laser irradiation of Foturan glass. (**a**,**b**) Optical images of the exposure map written on glass surface using 355 nm ps laser pulses (**a**) and corresponding critical fluence determination (**b**); (**c**,**d**) Optical images of the exposure map written on glass surface using 532 nm ps laser pulses (**c**) and corresponding critical fluence determination (**d**). The inset images represent detailed views of areas marked with square dots.

For each laser wavelength, several power densities were first applied in order to define the optimal power interval for the *D*_c_ evaluation. For a good statistical analysis, we have irradiated 25 identical points using the same laser power and the experiment was repeated twice. No visible differences were observed between experiments. In the case of UV, irradiation the laser power was systematically varied from 0.6 mW to 1 mW with 0.1 mW step (Figure 2a). The same procedure was used for the VIS wavelength for which the laser power was adjusted in the 30–50 mW range with a 5 mW step (Figure 2c).

After irradiation, the glass samples were submitted to a classical thermal treatment protocol described in the previous section. Upon the thermal treatment, brownish crystalline phase became visible on the samples by optical microscopy when the accumulated dose exceeded *D*_c_. The analyses of the irradiated patterns revealed a dose-dependent modification of samples for both laser wavelengths. We identified the irradiation parameters (power as a function of exposure time or number of pulses), at which the glass surface suffered visible modification by laser irradiation followed by thermal treatment (see stars marked in Figure 2a,c) for selective chemical etching, revealing *D*_c_. *F*_c_ is a function of the number of pulses for both 355 nm (Figure 2b) and 532 nm (Figure 2d) laser wavelengths. The fluences were estimated for each laser power considering the spot size of 4 µm in diameter. By linearly fitting the log-log representation data, in Figure 2b,d we determined the critical fluences of 1.742 J/cm^2^ (*m* = 3) for VIS and 0.635 J/cm^2^ (*m* = 2) for UV. By introducing these values into Equation (1), the critical dose values for both wavelengths were determined to be *D*_c_ = 5.28 J^3^/cm^6^ for VIS and *D*_c_ = 0.4032 J^2^/cm^4^ for UV, respectively. 

The evaluation of *D*_c_ is essential in order to further correlate with the translation stage programming for optimizing writing speeds of various complex 3D shapes. In addition, the subsequent wet chemical etching for the subtractive process is critically dependent on the laser irradiation dose for the high precision fabrication of embedded microfluidic channels.

### 3.2. Etching Rate Estimation

Using the estimated critical doses, we further determined the etching rate dependence for ps UV and VIS lasers, respectively. In HF solution, the contrast ratio in etching selectivity between the unexposed and the laser exposed Foturan glass was found to be 1:50, and was dependent on both the exposure dose and the HF concentration. This ratio was almost coincident with that determined when using the fs laser [24].

**Figure 3:**
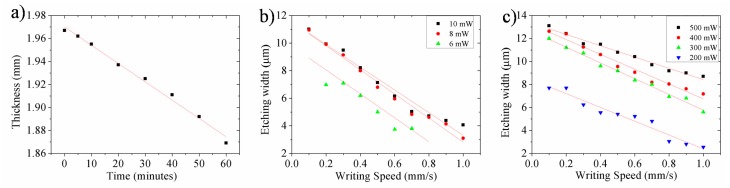
Etching of Foturan glass in 8% HF solution: (**a**) etched thickness as a function of etching time for unexposed glass; (**b**,**c**) width of open channels after 5 min of etching of the linear pattern written by UV (**b**) and VIS (**c**) lasers as a function of writing speed. Each linear regression curve corresponds to different laser power.

We have determined the etching rate of unexposed Foturan glass starting from an initial thickness of 2 mm after immersion in 48 mL solution of 8% HF concentration. At different time intervals, the glass was extracted from solution to measure its thickness with an electronic micrometer for up to 1 h of total exposure. The dependence of the glass thickness on the etching time is plotted in Figure 3a. The evaluation of the linear fitting evidenced an etching rate of about 1.6 µm/min which is in line with previously reported values, such as the study by Helvajian et al. (1.3 µm/min for a slightly lower concentration of 5% HF solution) [25].

The etching rates for VIS and UV lasers exposed samples were estimated by evaluating depths obtained after etching for different time intervals. In both cases, at moderate laser exposures (just above the modification thresholds), ~10 ± 2 µm/min etching rates were obtained. Consequently, the etching ratios were found to be at 1:10. These ratios are dependent on the laser exposure doses. Specifically, an etching rate of 20 µm/min can be achieved when increasing the laser power, corresponding to an etching ratio of up to 1:25. Comparing to etching ratios reported in other publications, where identical wavelength but different pulse widths were used to expose the glass, the values obtained in our study are similar while taking account of processing parameters such as irradiation doses and HF concentration.

The etching characteristics of glass modified by the high repetition rate ps laser were investigated by profilometry measurements. For both laser wavelengths, sets of five identical lines with 1 mm length and 100 µm interspace were written on the glass surface using different irradiation doses. To this purpose, predetermined scanning speeds ranging from 0.1 mm/s to 1 mm/s were employed at different laser powers, followed by annealing treatment. The channel widths created after 5 min of HF etching were evaluated, as presented in Figure 3b,c, for UV and VIS ps laser processing, respectively.

As a general observation, one can notice that the channel widths are dependent on the laser dose, as expected (Figure 3b,c). Indeed, for both wavelengths, a higher dose revealed the formation of wider channels (>10 µm), while only few micrometer widths are obtained for lower doses. In particular, one can obtain 11 µm width channels with UV ps laser pulses of 10 mW power at a writing speed of 0.1 mm/s, and narrower than 4 µm width, with 8 mW average laser power and 1 mm/s writing speed. Similarly, channels with widths wider than 12 µm are obtained by VIS ps laser pulses of 300 mW power and writing speed of 0.1 mm/s, and narrower than 3 µm width, at 200 mW laser power and 1 mm/s writing speed. These results allow us to predict the optimum laser processing parameters and scanning regimes for choosing appropriate interspaces between linear patterns in order to create more complex 3D structures. 

### 3.3. Fabrication of Microfluidic Embedded Channels

**Figure 4:**
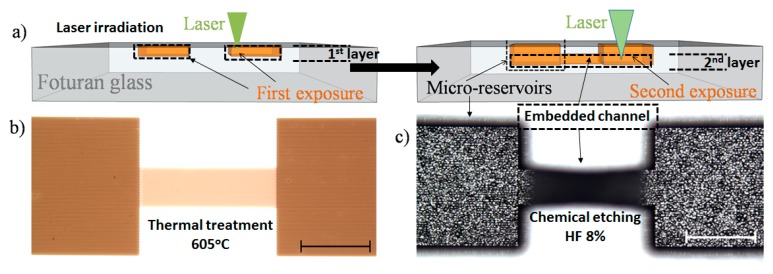
PLAE process of Foturan glass. (**a**) Sketch of the proposed design (with two open reservoirs connected by an embedded channel) and laser irradiation using two-layer configuration; and (**b**,**c**) partial view by optical microscopy of the obtained structure after irradiation with the VIS laser followed by (**b**) thermal treatment and (**c**) chemical etching in 8% HF solution. The scale bar represents 0.5 mm.

Further, the capability of generating embedded microfluidic channels in photosensitive glass using either UV or VIS high repetition rate ps laser pulses was demonstrated. By the new PLAE technique, we propose fabricating a simple microfluidic structure consisting of two open micro-reservoirs connected by an embedded channel (Figure 4). Specifically, square reservoirs of 1 × 1 mm^2^ area are connected by a 1 mm length and 300 µm width channel. The whole structure was designed and fabricated by irradiating the glass in a two-layer configuration (see Figure 4a): (*i*) the first layer (upper part) of the reservoirs (opening) were formed by scanning the focused laser beam on the glass surface and then (*ii*) the second layer (bottom part) of the reservoirs and connecting channel were formed by changing the laser focusing position from the glass surface into the volume at specific depths. Each layer consists of parallel lines written by linear scanning of the focused laser beam at a speed of 0.5 mm/s with lateral sliding at a 5 µm step between each line. The PLAE process was then followed by a two-step treatment at 500 °C for one hour followed by one at 605 °C for another hour (Figure 4b) and successive chemical etching in HF (Figure 4c) in order to obtain the designed structure. The etching time depends on the exposure parameters, which were varied between 50 and 60 min, similar with times used for FLAE.

**Figure 5:**
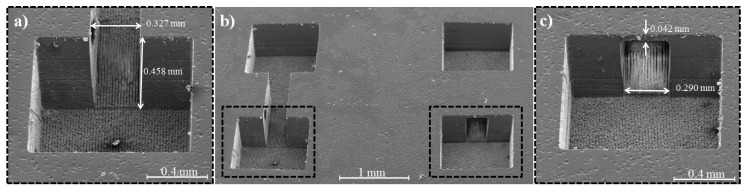
SEM images of structures fabricated by VIS-PLAE using a ps laser at power of 500 mW and a design of 10 μm interline spaces, at two different scanning speeds: 0.1 mm/s (**a**,**b**) and 0.9 mm/s (**b**,**c**). The laser irradiation was carried out with two-layer configuration: on the surface (first layer) and at 250 μm depth (second layer).

We created two structures written with the identical writing scheme using VIS-PLAE at 500 mW laser power, 1 µJ energy, and 10 µm interline spaces, but at different scanning speeds to investigate influence of the exposure dose on the created structures. The laser irradiation was carried out on the surface (first layer) and at 250 µm depth (second layer) with writing speeds fixed at 0.1 mm/s (Figure 5a,b) and 0.9 mm/s (Figure 5c) respectively. SEM analyses have revealed that a lower irradiation dose (correlated with faster writing speed and smaller number of applied laser pulses) can generate embedded microfluidic channels in glass volume after 50 min of wet chemical etching (Figure 5b,c). Contrarily, when slower speed (higher irradiation dose) is applied, the channel rooftop disappeared due to second layer overexposure (Figure 5a). Indeed, by increasing the irradiation dose inside glass, the volume affected by multiphoton absorption becomes larger. Consequently, if the position of the second layer is close enough to the surface, the channel rooftop can be removed during the chemical etching. It is worth mentioning that fabrication of the structure obtained at the scanning speed of 0.1 mm/s (Figure 5a) required a processing time of about 70 min, while the structure fabricated at 0.9 mm/s (Figure 5c) could be written in only 10 min. One may further adjust the thickness of channel rooftop by tailoring the scanning speed.

Fabrication of the similar structure was also attempted with the same writing scheme by ps laser processing at a wavelength of 355 nm. After finding optimal parameters, embedded channels could be also fabricated by PLAE using a UV ps laser (UV-PLAE). In particular, by applying a power of 12 mW, energy of 0.024 µJ, a 5 µm interline step and a writing speed of 0.5 mm/s the structure with two open reservoirs connected by embedded channel was successfully created. With these processing conditions, the entire structure was written in less than 20 min and an etching time of 60 min.

**Figure 6:**
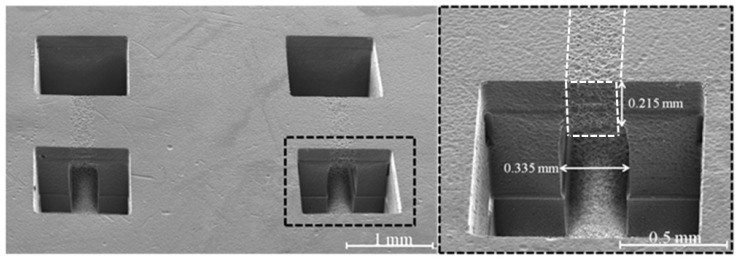
SEM images of twin structures fabricated by UV-PLAE using a ps laser of 12 mW laser power and 5 μm interline spaces, at 0.5 mm/s scanning speed. The laser irradiation was carried out using two-layer configuration: (on the surface (first layer) and at 500 μm depth (second layer)).

In Figure 6, we present SEM images of twin structures separated by 2 mm written in Foturan glass by UV-PLAE. Pairs of two open square reservoirs of 1 × 1 mm^2^ area are connected by 1 mm length and 335 µm width embedded channels.

## 4. Discussion

During interaction with solid materials, the pulsed laser beam is depositing its energy, inducing different phenomena dependent on the pulse energy, duration, and focusing optics [26]. It is rather a thermal process for long pulses (nanosecond regime), while a physical aspect predominates for ultrashort pulses (less than a few ps pulses that are shorter than electron-phonon coupling time in materials) [27]. Consequently, in the case of ultrashort pulses, the heat-affected zone is minimized [20,28,29]. In most of the studies, the photo-physical and photo-chemical mechanisms involved during glass processing with ultrashort pulses are evidenced for femtosecond laser pulses and extensively addressed, from both fundamental and applicative points of view [12,30,31,32]. Origins of material modification under interaction with fs pulses could be related to the densification induced by pressure wave and/or fast heating-cooling processes [33,34]. As a result, electrons in the conduction band are heated by the laser pulse very quickly so that they do not have time to diffuse out from the irradiated volume or to recombine. The photoionization is then responsible of seeding electrons for the subsequent avalanche ionization. It was found out that the electron density increases by avalanche ionization until its plasma frequency reaches the critical plasma density [35]. On the other hand, the avalanche ionization is more efficient for longer pulses since it allows more time for increasing the electron density. In our study, ps laser irradiance values of 3.73 × 10^13^ and of 3.57 × 10^12^ Wcm^−2^ for VIS and UV, respectively, were determined, below values of 10^14^ Wcm^−2^ for fs laser irradiances [16]. These values for ps laser irradiances corresponds to a Keldysh parameter above 1.5 which indicates the multi-photon absorption is more dominant rather than tunneling ionization in the case of both UV and VIS irradiation [36]. In contrast, pulses longer than a few tens of ps do not reach enough intensity to directly photoionize the electrons.

In the first studies using ultrafast laser reported in literature, Foturan photosensitive glasses were exposed to 150 fs laser pulses at 775 nm wavelength [16]. A second thermal treatment step followed by isotropic etching conducted to a preferential material removal from irradiated regions. In this case, the experimental investigation of *F*_c_ on the number of pulses allowed the calculation of a critical dose of *D_c_* = 1.3 × 10^−5^ J^6^/cm^12^ with *m* = 6 for the 775 nm wavelength fs laser. Interaction mechanisms of laser pulses with Foturan glass were further explored by employing: (*i*) fs laser (150 fs, 775 nm, 1 kHz), and ns lasers of (*ii*) 266 nm; (*iii*) 355 nm; and (*iv*) 308 nm laser wavelengths [17]. A significant increase in absorption spectrum of the exposed samples around 360 nm was found for fs irradiated glasses corresponding to absorption from oxygen deficient centers which originated from the interband excitation of electrons. The absorption at 315 nm related to Ce ions was not observed, suggesting that Ce^3+^ ions do not contribute to electron generation for the reduction to Ag atoms in the case of fs-irradiated samples at 775 nm. Thus, it was concluded that free electrons were generated by two-step interband excitation through the defect levels with three-photon absorption each resulting in six photons in total for photoreaction. A similar photoreaction mechanism by successive interband excitation was evidenced for 266 nm ns laser-irradiated samples, but by a linear two-photon process, i.e., two-step excitation by single-photon absorption each. On the other hand, in the case of 355 nm laser-irradiated samples, free electrons are generated by Ce^3+^, while in the case of the 308 nm laser both absorption by Ce^3+^ (single-photon absorption) and interband excitation (the linear two-photon process) was found. In this study, we evaluated the possibility to obtain embedded microfluidic channels in Foturan glass by using a high repetition rate (500 kHz) laser of <10 ps pulse duration at 532 nm and 355 nm wavelengths. A nonlinear absorption process is evident at the interaction of both 532 nm and 355 nm laser pulses with Foturan glass, since the absorption edge of Foturan is shorter than 350 nm. The Foturan glass also has an absorption peak around 315 nm ascribed to Ce^3+^ absorption, which corresponds to photon energy of 3.93 eV. Therefore, for 532 nm with the photon energy of 2.33 eV, two photons are necessary to generate free electrons from Ce^3+^. Another channel for free electron generation is interband excitation, for which the two-step excitation model through the intermediate state has been proposed [17]. For this excitation, the photon energy of 3.49–4.66 eV is required for each step, indicating four photons in total for the 532 nm beam. We have found *m* = 3 photon process for VIS pulses. Thus, it is likely that the free electrons are generated by both the Ce^3+^ absorption and the interband excitation through the intermediate state similarly to the case of the 308 nm ns laser [17]. In the case of UV pulses, at 355 nm, with the photon energy of 3.49 eV, we found *m* = 2 photon process which should correspond to free electron generation by two-photon absorption by Ce^3+^ similarly to the case of the 355 nm ns laser [17]. In addition, we consider that, in our experimental conditions, the high repetition rate of the ps laser pulses can increase the nonlinear absorptivity into Foturan glass material [37]. The alteration of the surface and volume morphologies that can be observed above the embedded channel in Figure 6 (white dotted regions) may support the hypothesis that, for high repetition rate UV ps laser pulses, the heat effect becomes more important when the laser beam is focused above a critical intensity. 

During interaction with a Gaussian laser beam (fs or ps durations) with Foturan glass, the absorption profile affects the shape of modification volume to develop an elliptical cross-sectional shape of the crystallized area. In case of a high intensity fs laser pulse, the main advantage resides in high density, compact modification at very low power, which can be used for high resolution micro-processing. On the other hand, ps lasers can represent a viable alternative for large-scale processing both on surface and in volume due to high average power compensation since longer, high-energy, pulses can modify more material per pulse. Indeed, a larger energy can be deposited in the material since the time is longer. Thus, this energy allows more time for growth of electron density during the laser irradiation and in consequence an increase of formed Ag atoms. These Ag atoms are then responsible of the formation of larger Ag clusters and a larger glass crystalline area during thermal treatment. As a result, one may consider that the area of the crystalline phase is dependent on pulse duration. Using the same focusing optics, ps laser beams can consequently decrease the processing times as compared with femtosecond lasers. As a direct comparison, to create a similar structure, irradiation time was 1.5 h for laser pulses of 360 fs at 522 nm (2 mm/s scanning speed and 250 KHz laser repetition rate) while, in our case, it was of approximately 10 min at a scanning speed of 0.9 mm/s and 500 KHz (SI in [38]). On the other hand, in case of shorter pulses one needs less energy to achieve the intensity for optical breakdown allowing the achievement more precise machining with femtosecond lasers rather than the longer pulse lasers. Thus, higher resolution processing is achieved with fs pulses than ps pulses. In case of ps laser pulses, a more efficient process could be achieved for UV pulses since the critical dose is one order of magnitude less than in case of VIS pulses compensating the laser power conversion efficiency (1:2 in case of VIS and 1:3 for UV). 

The high repetition rate ps laser pulses could thus stand as a prospective processing benefit, attractive for several applications that require high speed and cost-effective manufacturing. On the other hand, by controlling the irradiation dose with respect to the laser wavelength and etching parameters one can explore the unique characteristics in 3D microfabrication for a wide range of applications.

We have finally demonstrated that PLAE is a suitable and very fast fabrication method of 3D embedded microfluidic channels with good aspect ratio and sharp edges without any cracks by using either ps laser pulses in UV or VIS.

## 5. Conclusions

High repetition rate ps laser processing at both 532 and 355 nm wavelengths was applied for fabrication of 3D microfluidic structures in Foturan glass. A three-photon process with a critical fluence of 1.742 J/cm^2^ for the VIS case and a two-photon process with 0.635 J/cm^2^ for the UV case were found. Critical dose values of 5.28 J^3^/cm^6^ for VIS and 0.4032 J^2^/cm^4^ for UV cases were calculated. 

Straight lines of 1 mm length and 100 µm interspace were then written on the glass surface at different scanning speeds using different laser powers. Open channels with widths ranging from 3 to 13 µm were developed by thermal treatment and HF etching depending on irradiation doses. Based on a subtractive fabrication process consisting of selective removal of laser-modified regions by chemical etching, we could further fabricate 3D hollow structures in glass by PLAE at both 355 nm and 532 nm laser wavelengths. Due to high power, high repetition rate laser pulses which increase multiphoton absorption, we could apply the laser irradiation process very fast for fabrication of embedded structures. A simple configuration consisting of two micro-reservoirs connected by an embedded channel can be achieved in less than 10 min of laser irradiation by the PLAE technique using either 355 or 532 nm wavelengths.
